# HPV genotype prevalence and distribution during 2009–2018 in Xinjiang, China: baseline surveys prior to mass HPV vaccination

**DOI:** 10.1186/s12905-019-0785-3

**Published:** 2019-07-08

**Authors:** Jing Wang, Dandan Tang, Kai Wang, Jialu Wang, Zhaoxia Zhang, Yanxia Chen, Xueliang Zhang, Cailing Ma

**Affiliations:** 1 State Key Laboratory of PPTHIDCA(Pathogenesis, Prevention and Treatment of High Incidence Diseases in Central Asia) / Department of Gynecology, First Affiliated Hospital of Xinjiang Medical University, Urumqi, Xinjiang China; 2 College of Public Health, Xinjiang Medical University, Urumqi, China; 3 Department for College of Medical Engineering and Technology, Xinjiang Medical University, Urumqi, Xinjiang China; 4 Department of Medical laboratory, First Affiliated Hospital of Xinjiang Medical University, Urumqi, Xinjiang China

**Keywords:** Human papillomaviru, Genotype distribution, Xinjiang region, HPV vaccination

## Abstract

**Background:**

The aim of this paper was to conduct a baseline survey of HPV infection in unvaccinated women in Xinjiang Uyghur Autonomous Region before the mass use of HPV vaccine.

**Methods:**

Between 2008 and 2018, the HPV genotype detected by a PCR-based hybridization gene chip assay of 37,722 women who were from Gynecology Department and Health Management Center of the First Affiliated Hospital of Xinjiang Medical University were tested HPV genotype by a PCR-based hybridization gene chip assay. All statistical analysis methods were performed with this statistical software including Python version 3.6.1, R Software 3.5.1 and Excel 2011.

**Results:**

The total positive rate for HPV was 14.02%, the most prevalent genotypes were HPV 16 (3.79%), HPV 52 (2.47%), HPV 58 (1.76%), HPV 53 (1.35%) and HPV 31 (0.72%). The single infection (11.34%) and high-risk HPV (HR-HPV) infection (9.72%) was the main prevalence of HPV. Age-specific HPV distribution was presented as a bimodal curve, while the youngest age group (≤25 years) presented the highest HPV infection rate (20.78%), which was followed by a second peak for the 36–40 age group. According to the ethnic stratification, the HPV infection prevalence ranging from the high to low was: Mongol (16.36%), Hui (15.15%), Kazak (14.47%), Han (14.43%), Other (14.37%), Uygher (10.96%). From 2009 to 2013, the HPV infection rate fluctuated but did not changed much. It peaked in 2014 and then fell significantly, reached the bottom point in 2017 and rose slightly in 2018. In 2015, the infection rate of HPVl6 and 52 in the population was almost the same (both 3.40%) the infection rate of HPV52 type (3.31%) was higher than that of HPVl6 type (2.18%) and became the dominant type in 2016.

**Conclusions:**

We present data regarding the prevalence and type distribution of HPV infection, which could serve as the valuable reference to guide nationwide cervical cancer screening. These baseline data enable the estimates of maximum HPV vaccine impact across time and provide critical reference measurements which are important to the assess of clinical benefits and potential harms in HPV vaccination and the increase in non-vaccine HPV types.

## Background

Cervical cancer (CC) is the fourth most common cancer in women worldwide. Every year, approximately 570,000 new cases and 310,000 deaths of CC are reported around the world, 85% of which have taken place in developing countries [1]. As the most populous developing country, China faces serious burden of cervical cancer. According to the latest cancer statistics from the national cancer center, there were 98,900 new cases of cervical cancer in China in 2015, with an incidence rate of 6.25%, up 0.21 percentage points from 6.04% in 2014. There were 30,500 deaths with a mortality rate of 3.96%. The cervical cancer incidence rate of different regions in China from high to low was: the central, the western and the eastern region. The mortality rate in the western region is slightly higher than that in the central region, and the eastern region is the lowest, which is related to the low prevalence of cervical cancer screening and high HPV infection rate in less developed regions. Xinjiang is located in the northwest of China, the incidence of cervical cancer has been at a high level. Screening results have indicated that the incidence and mortality rate of cervical cancer are 459–590/100,000 and 17.78/100,000, respectively, for women who live in Xinjiang [2]. These numbers are obviously higher than those reported for other ethnic groups.

Human papillomavirus (HPV) infection is the most common sexually transmitted viral infection worldwide, with approximately 75% of sexually active men and women exposed to HPV during their lives [3]. HPV is an etiologic factor for the malignant lesions of cervix, vagina, vulva, and penis, and genital warts [4]. According to a report of the International HPV Reference Center, the HPV family is made up of 202 different genotypes [5]. Among the 202 patients, about 40 are found in the female genital tract [6] and could be divided. According to their ability to produce malignancy, they were classified as high-risk types (HR-HPV), which might lead to carcinogenic progression of lesions (e.g. HPV 16 and 18) [7], and low-risk types (LR-HPV) (e.g. HPV 6 and 11), in which the high grade cervical lesions (HSIL) were rarely found, but produced the majority of genital warts [8, 9].

The discovery that the persistent HR-HPV infection causes cervical cancer and the HPV is a necessary cause of cervical cancer [10, 11] has changed the global strategy for cervical cancer prevention in the twenty-first century. Because of the accumulated knowledge of the causal relationship between HPV infection and CC over the past three decades, the detection of the viral DNA became an attractive approach to identify women at risk for developing CC [12, 13]. HPV DNA testing is widely used around the world as one of the main methods for secondary prevention of cervical cancer. Primary prevention could be achieved by injecting HPV preventive vaccine. With the development of research, it has been found that prophylactic HPV vaccination could effectively prevent the occurrence of precancerous lesions and cervical cancer. Estimation of pre-immunization prevalence of HPV and distribution of HPV types are the fundamental to understand the subsequent impact of HPV vaccination. A growing number of studies have been investigated into the prevalence and genotype distribution of HPV in many geographical regions and the results vary considerably [14–17]. Knowledge on the distribution of individual HPV types in different geographical areas is essential for the optimization of cervical cancer preventive strategies such as vaccination and HPV-DNA primary screening [18].

Cervical cancer vaccines have been available in more than 130 countries and regions around the world over the past decades and are widely vaccinated among females. Based on good clinical protection and safety data from HPV vaccines, the world health organization (WHO) has also issued recommendations to encourage the use of HPV vaccines in the appropriate population to reduce the incidence of cervical cancer. Data released by the National Bureau of Statistics of China showed that the total population of the mainland was 1.375 billion at the end of 2015, among which the female population was 670,480,000(48.76%), the proportion of women aged 0–45 was about 84.02%, suggesting that a large number of women were at an age appropriate for HPV vaccination. The first approved HPV vaccine was officially launched on July 31, 2017 in China. At present, only a small number of females of the right age have been vaccinated against HPV-related disease in China, especially in Xinjiang. Up to now, there was no large sample study on the genotype prevalence of HPV in Xinjiang. The aim of this paper was to conduct a baseline survey of HPV infection in women in Xinjiang before the mass use of HPV vaccine. It would lay a foundation for the observation of the efficacy of HPV vaccine and HPV genotype change after vaccination in the future.

## Methods

### Study participants and setting

A total of 37,722 females aged 13–89 years were enrolled in this study from November 2008 to July 2018. All participants were from Gynecology Department and Health Management Center of the First Affiliated Hospital of Xinjiang Medical University. The patients visited the hospital for various reasons, including physical examination, infertility, vaginitis, cervicitis, undiagnosed abdominal pain, genital warts and cervical intraepithelial neoplasia. Clinical data were collected from the participants, and a molecular survey of HPVs was conducted. All the women enrolled in this study had sexual life history, and the study excluded those who were pregnant at the time of enrollment, who had been vaccinated against HPV, who had undergone hysterectomy, who suffered from cervical cancer and received radiation and chemotherapy, and who had undergone colposcopy in the 24 months prior to the enrollment. All participants were told to refrain from sexual activity and avoid washing the genitals for 48 h before sample collection. Eligible women were included in the study after signing an informed consent form.

Quality control of HPV genotyping and data input procedures were implemented throughout the study, which was conducted in accordance with the Good Epidemiological Practice Guidelines and the Principles of the Declaration of Helsinki and was approved by First Affiliated Hospital of Xinjiang Medical University ethics committee. Quality control of HPV genotyping included sample quality requirements, test quality requirements, test report entry and issuance, and molecular diagnostic item analysis performance standards. The sample container was a disposable HPV collection tube. When receiving the sample, there was a clear identification and the uniqueness of identification was ensured. The sample for secondary testing could be traced back to the original sample, and the label on the sample was consistent with the label on the application form. Samples were rejected under the following circumstances: if the application form was filled with illegible handwriting or there was doubt, the sample storage solution was less than 1 ml, and the volume of the sample transferred to the secondary catheter was less than 1 ml. The original sample, nucleic acid extractive and/or nucleic acid amplification products stored in − 80 °C refrigerator, were used for recheck. Test Quality included External Quality Assessment (EQA) and Internal Quality Control (IQC). HPV test in our hospital participated in the quality evaluation system of the clinical test center of the national health and family planning commission, and all of them were qualified. Qualified laboratory staff operate were in strict accordance with standard operating procedures, and weakly positive and negative quality control products were randomly placed among clinical samples for each test to monitor the accuracy of the experiment. Laboratory staff recorded the experiment process in detail, analyzed the quality control results, if the results were out of control, the reasons for the control would be judged, the corrective measures would be taken, and then the corrective results would be evaluated. The person in charge of the laboratory should regularly supervise the results of internal quality control. When the experimental data were input, double check was used. The original experimental records were retained for each experiment. For the failed experiment and unsatisfactory results, the reasons were analyzed and the recheck was conducted. Molecular diagnostic item analysis performance standards: After equipment fault repair, analysis system comparison: 5 samples, covering the test interval, at least 4 samples measurement results bias<±7.5%. Retention sample retest criteria: according to the stability requirements of the project, the longest duration samples were selected, with 5 samples covering the test interval and at least 4 samples with the test result bias<±7.5%. Test criteria for inter-batch difference of reagent: 5 samples tested with old batch number, covering measurement interval (including negative, critical, low, medium and high values), and at least 4 samples with test result bias<±7.5%.

### Sample collection and management

After the cervix uteri were fully exposed with the aid of a vaginal speculum, a specially designed cervical brush (known as a cytobrush) gently scoured the cervix uteri through clockwise 5 rotations to obtain the cervical secretions and cells needed for further testing. The samples were placed in a cell preservation solution and stored at 2–8 °C until HPV DNA extraction and genotyping could be performed. The HPV genotype testing of the samples was completed within a week.

The transfer of samples is the responsibility of the nursing staff, the hospital is equipped with a portable thermostat to facilitate the transfer of special specimens, HPV typing test samples belong to this kind of special specimens.Our process was that the clinician taked the samples and placed the samples in the refrigerator of the department (2–8 °C), and then the nurse placed the samples in a portable thermostat (2–8 °C), finally the samples were put in the laboratory refrigerator. Since 2008, our hospital began to carry out HPV typing detection. This study retrospectively analyzed all patient data in the existing database that were complete and available.

### HPV DNA extraction, PCR amplification and genotyping

This part of methodology has been elaborated in my previous articles [19] and will not be repeated here. DNA was extracted by magnetic bead method, and the negative and positive quality control products were extracted throughout the whole process. After the extraction was completed, the concentration and purity of nucleic acid were tested by ultraviolet spectrophotometer. Quality control in genotyping detection, on the one hand, negative and positive quality control was added, on the other hand, the microarray for detecting the chip contained five QC.BC.PC.NC.IC quality control points.

### Statistical analysis

Analyses were done with Python (version 3.6.1), R Software (version 3.5.1) and Excel (version 2011). Descriptive statistical analysis was performed on the distribution of HPV genotypes using indicators such as frequency and prevalence. Chi square testing was used to test for the differences in HPV prevalence between simple infection and multiple infection in different age groups, time groups, and ethnic groups. Chi square tests were also conducted to test for differences in HPV prevalence among HR HPV infection, LR HPV infection and Mixed HPV infection in different age groups, time groups, and ethnic groups.

## Result

### Prevalence and genotype distribution of HPV

Through nearly a decade of surveillance, a total of 37,722 objects’ samples were collected and detected by HPV genotype. The genotype test showed 5287 samples were HPV positive, so the overall prevalence of HPV in the study population was 14.02% for all types**.**

**Fig. 1:**
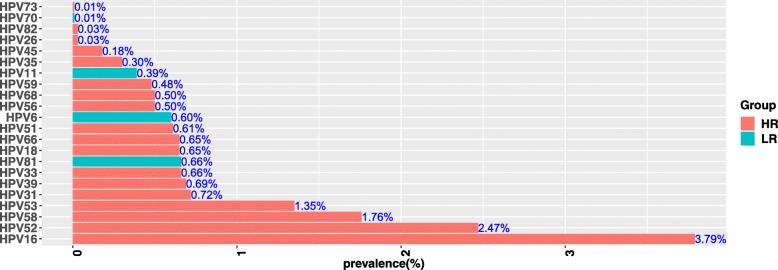
Prevalence and genotype distribution of HPV (HR-HPV and LR-HPV)

The details of genotypes distribution of HR-HPV and LR-HPV were shown in Table 1 and Fig. 1, the most common 10 genotypes of HPV infection were as follows: HPV 16 (1462, 3.79%), HPV 52 (953, 2.47%), HPV 58 (680, 1.76%), HPV 53 (520, 1.35%), HPV 31 (276, 0.72%), HPV 39 (264, 0.69%), HPV 33 (254, 0.66%), HPV 81 (253, 0.66%), HPV 18 (249, 0.65%), and HPV 66 (249, 0.65%). Notably, HPV 16 was the most common genotypes among all the patients. Besides, HPV 16, 52, 58 were the top three prevalent genotypes among HR-HPV and HPV 81, 6, 11 were the top three prevalent genotypes among LR-HPV.

**Table 1:** Frequency and prevalence of genotype of HPV among women

Genotype	Frequency	Prevalence
16	1462	3.79%
18	249	0.65%
26	10	0.03%
31	276	0.72%
33	254	0.66%
35	115	0.30%
39	264	0.69%
45	71	0.18%
51	235	0.61%
52	953	2.47%
53	520	1.35%
56	192	0.50%
58	680	1.76%
59	185	0.48%
66	249	0.65%
68	193	0.50%
73	4	0.01%
82	11	0.03%
6	230	0.60%
11	151	0.39%
70	3	0.01%
81	253	0.66%

### Prevalence of single and multiple HPV infection

As shown in Table 2, single infection (4278 cases, 11.34%) was the most common form among the infected cases while multiple infections (1009 cases, 2.67%) were the rare form. Moreover, among multiple infections, the prevalence of HPV decreased significantly as the number of infected genotypes increased, with 841 (2.18%) patients of double infection, 97 (0.25%) patients of triple infection, 58 (0.15%) patients of quadruple infection, 6 (0.02%) patients of quintet infection, and 7 (0.02%) patients of sextuple infection. The main prevalence was HR-HPV infection, while the pure HR-HPV infections (including single and multiple HR-HPV infection) were 3668 (9.72%) cases, the pure LR-HPV infections (including single and multiple LR-HPV infection) were 1239 (3.28%) cases, and the mixed infections (including HR-HPV and LR-HPV mixed infection) were 360 (0.95%) cases.

**Table 2:** Prevalence of single and multiple HPV infection

Genotype	Frequency	Prevalence
Single	4278	11.10%
Double	841	2.18%
Triple	97	0.25%
Quadruple	58	0.15%
Quintet	6	0.02%
Sextuple	7	0.02%

### Prevalence of HPV grouped by age

Overall, 37,722 women (age 13–89 years) were included in this study. All the participants were divided into eight groups ranging from ≤25 years, 26–30 years, 31–35 years, 36–40 years, 41–45 years, 46–50 years, 51–55 years, and ≥ 56 years. Among the eight age groups, there were significant differences in the distribution of pure HR, pure LR and mixed HPV infection (*P* < 0.05), while there were no statistical differences in the distribution of single and multiple infections (*P* > 0.05), relevant data could be seen in the Table 5.

**Fig. 2:**
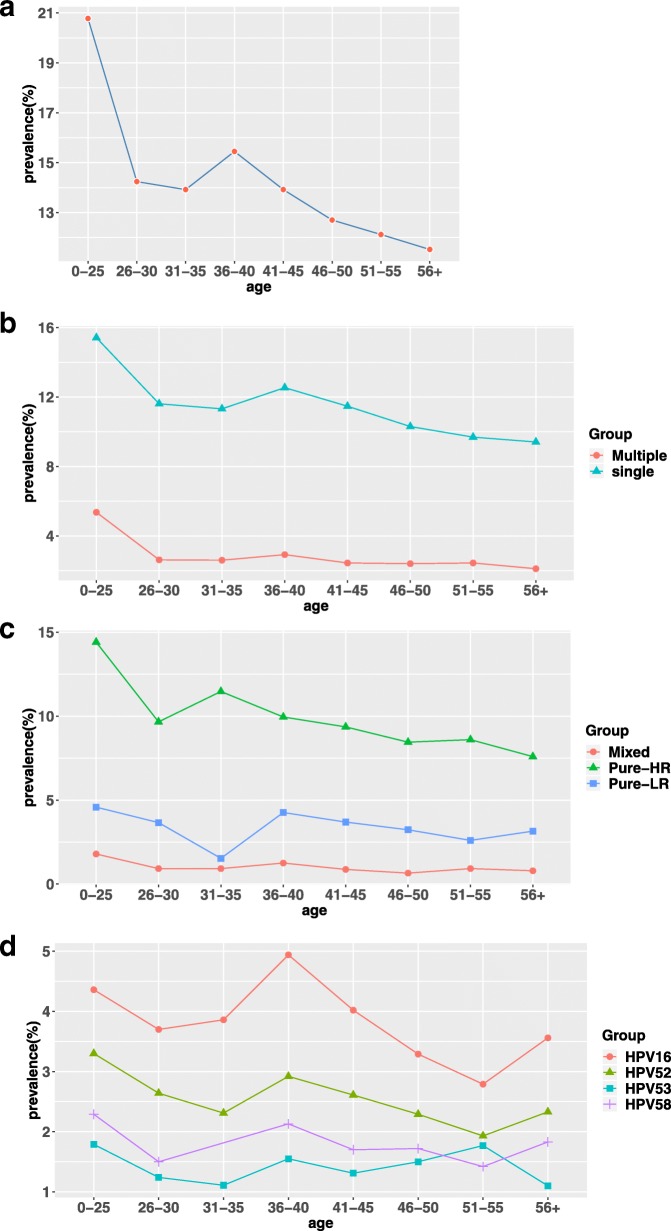
Prevalence of HPV grouped by age: (**a**) prevalence of all HPVs infection in each age group; (**b**) prevalence of single and multiple infection of HPV in each age group; (**c**) prevalence of high-risk (HR), low-risk (LR), and mixed HPV infection in each age group; (**d**) prevalence of HPV 16, 52, 53, and 58 in each age group

Age-specific HPV distribution presented as either a bimodal curve, the first peak appeared in women aged ≤25 years (20.78%), however, the HPV prevalence dropped drastically after the first peak, maintained a plateau for 10 years before reaching the second peak (15.45%) at 36–40 age group, then followed by a moderately decline, finally reached the bottom at ≥56 age group (11.52%) (Fig. 2a). After stratification by age, single HPV infection was still dominant in all age groups, and the change trend was consistent with the overall trend. The multiple infection form was less than single infection, the peak of multiple HPV infection occurred in women aged ≤25 years, and then remained very low level stably at all ages without significant fluctuations (Fig. 2b). Figure 2c indicated the distribution and change trend of pure high-risk and pure low-risk HPV types and mixed HPV types by age. Pure high-risk HPV infection still took up the majority, and its variation trend was similar to that of the overall and single HPV infection. The difference was that between the ages of 31 and 35, pure high-risk HPV infection was the second peak while pure low-risk HPV infection hit a trough. The lowest point of pure high-risk HPV infection was found at ≥56 age group while pure low-risk infection increased slightly. The mixed infection rate was lower in all age group and the highest rate was still in the population aged ≤25 years. Figure 2d revealed the prevalence and variation tendency of the four major HPV genotypes (16, 52, 58, 53) among different age groups in this study. The prevalence of HPV 16 was much higher than that of HPV 52, 58 and 53 in every age group, and they all had a similar trend before 40 years old. The first peak appeared at ≤25 age group, then gradually decreased, and the second peak appeared at 36–40 age group. The first peak of HPV16 was lower than the second peak, on the contrary, the first peak of remaining three types was higher than the second peak. In the 40–50 age group, HPV16, 52, 58 infection rates gradually decreased, reached the lowest point at the 51–55 age group, and then marginally increased. HPV53 infections, by contrast, rose slowly after age 40, reached another peak in the 51–55 years, followed by a gradually declined.

### The distribution of HPV genotypes in different nationalities

**Fig. 3:**
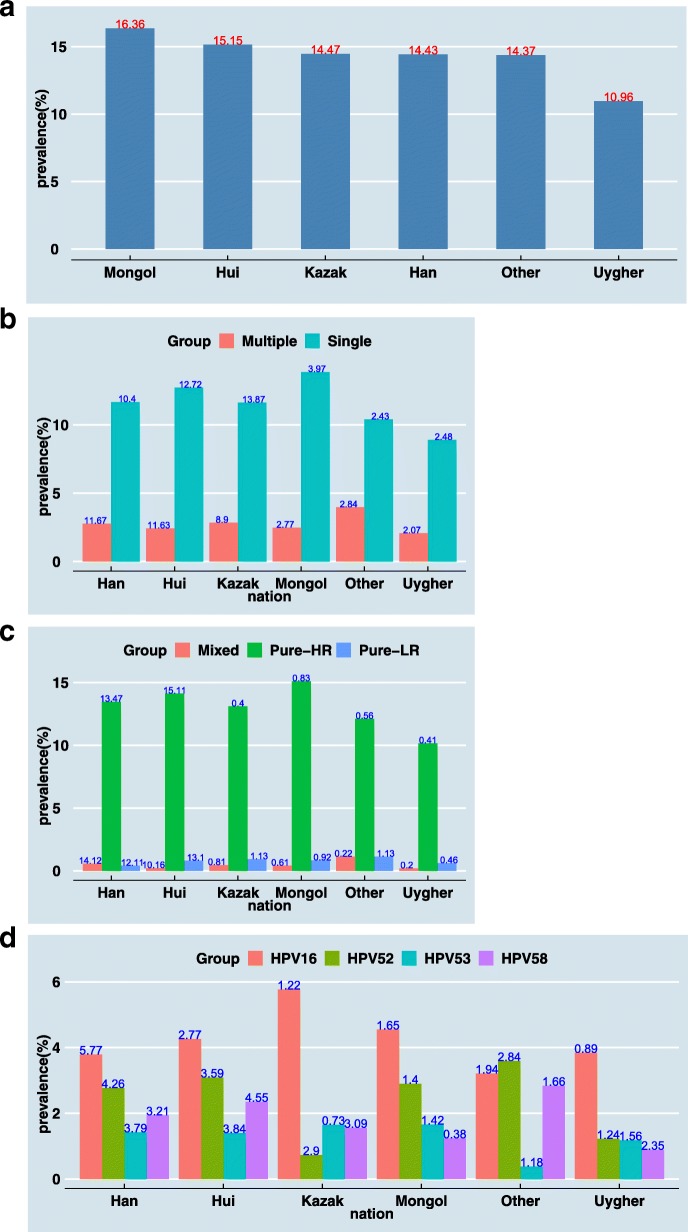
Prevalence of HPV grouped by nationality: **a** prevalence of all HPVs infection in each nationality group; **b** prevalence of single and multiple infection of HPV in each nationality group; **c** prevalence of high-risk (HR), low-risk (LR), and mixed HPV infection in each nationality group; **d** prevalence of HPV 16, 52, 53, and 58 in each nationality group

Xinjiang was a multi-ethnic region with 47 ethnic compositions and was mainly inhabited by Han, Uygur, Kazak, Hui, Mongol, Kirgiz, Xibe, Tajik, Uzbek, Manchu, Daur, Tatar, Russia and so on. Xinjiang was one of China’s five ethnic autonomous regions. Due to the limited population of other ethnic groups, the distribution differences of HPV positive rate among different ethnic groups were listed separately among the five major ethnic groups of Han, Uygur, Kazak, Hui, and Mongol, while other ethnic groups were merged into one data for analysis. The differences of HPV prevalence among nationalities were shown in Tables 3, 5, 6 and Fig. 3. Among the six nationalities groups, there were significant differences in the distribution of pure HR, pure LR and mixed HPV infection (*P* < 0.05), while there were no statistical differences in the distribution of single and multiple infections (*P* > 0.05) (Table 5).

**Table 3:** The distribution of HPV genotypes in different nationalities

Nationality	Number of patients	Frequency	Prevalence
Han	29,178	4211	14.43%
Uygher	5080	557	10.96%
Kazak	1092	158	14.47%
Hui	1360	206	15.15%
Mongol	483	79	16.36%
Other	529	76	14.37%

Most of the screening population was Han, the prevalence of HPV infection ranged from high to low was: Mongol, Hui, Kazak, Han, Other, Uygher (Fig. 3a). All ethnic groups were dominated by single infection, of which Mongol’s single infection was still the highest, and the rate of multiple infections in other group was higher than that of the remaining ethnic groups (Fig. 3b). Pure HR-HPV infection was higher than pure LR and mixed HPV infection in all ethnic groups (Fig. 3c). HPV16 had the highest infection rate among all ethnic groups, while in other groups HPV52 infection had the highest rate. Compared with other ethnic groups, Kazak had the highest HPV16 infection rate and the lowest HPV53 infection rate (Fig. 3d).

### Prevalence of HPV grouped by year

**Fig. 4:**
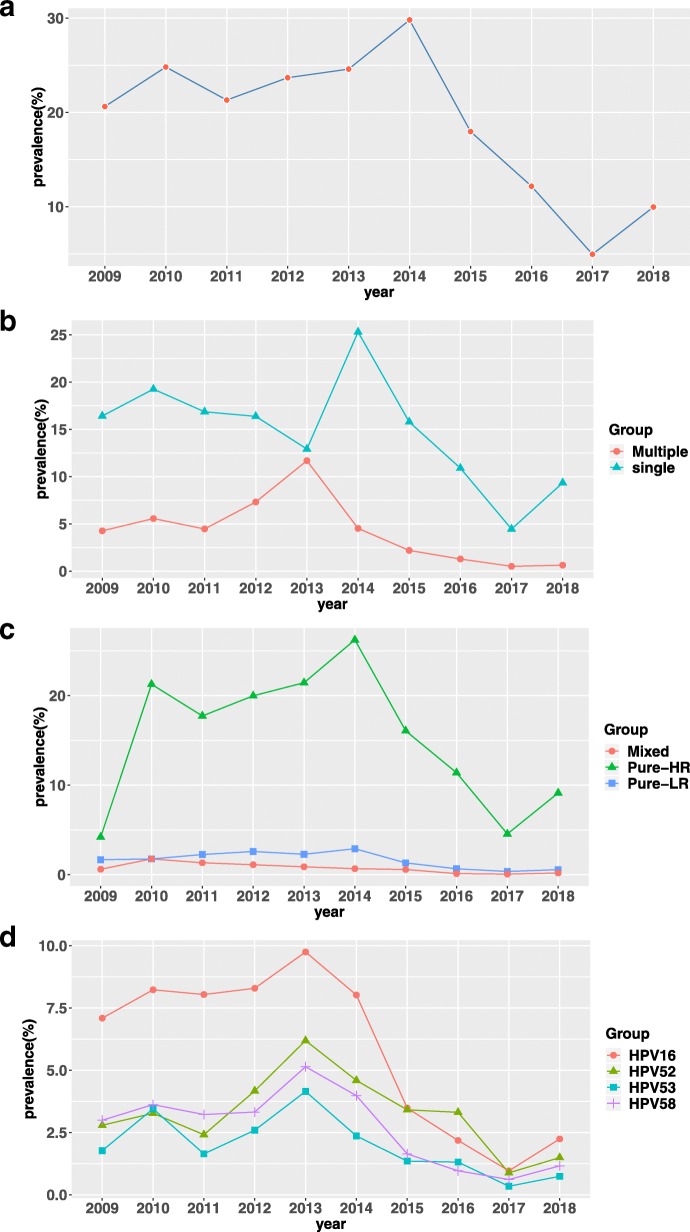
Prevalence of HPV grouped by year: (**a**) prevalence of all HPVs infection in each year group; (**b**) prevalence of single and multiple infection of HPV in each year group; (**c**) prevalence of high-risk (HR), low-risk (LR), and mixed HPV infection in each year group; (**d**) prevalence of HPV 16, 52, 53, and 58 in each year group

The total data of this study ranged from November 1, 2008 to July 31, 2018. As there were only 2 months of data in 2008, we got rid of that data when the annual changes of HPV prevalence was analyzed. In 2018, we collected data for 8 months, so we analyzed this part of the data to summary the results. The relevant data were shown in Tables 4, 5 and 6 and Fig. 4. Significant differences were found among different years’ groups in terms of simple and multiple infection (*P* < 0.05), pure HR, LR, and mixed HPV infection (*P* < 0.05) (Table 5).

**Table 4:** Prevalence of HPV grouped by year

Year	Number of patients	Prevalence
2009	1974	407(20.62%)
2010	2322	576(24.81%)
2011	1952	416(21.31%)
2012	2352	557(23.68%)
2013	1712	421(24.59%)
2014	1483	442(29.80%)
2015	3106	558(17.97%)
2016	6657	810(12.17%)
2017	10,693	530(4.96%)
2018	5366	535(9.97%)

**Table 5:** Comparison of single and multiple infection, HR and LR HPV infection grouped by age, nationality and year

	Total	Single	Multiple	Pure-HR	Pure-LR	Mixed
Age						
≤ 25	1790	276 (15.42%)	96 (5.36%)	258 (14.41%)	82 (4.58%)	32 (1.79%)
26–30	4917	571 (11.61%)	129 (2.62%)	475 (9.66%)	180 (3.66%)	45 (0.92%)
31–35	5877	665 (11.32%)	153 (2.60%)	674 (11.47%)	90 (1.53%)	54 (0.92%)
36–40	6342	795 (12.54%)	185 (2.92%)	631 (9.95%)	270 (4.26%)	79 (1.25%)
41–45	7042	808 (11.47%)	172 (2.44%)	659 (9.36%)	260 (3.69%)	61 (0.87%)
46–50	5418	558 (10.30%)	130 (2.40%)	458 (8.45%)	175 (3.23%)	35(0.65%)
51–55	3159	306 (9.69%)	77 (2.44%)	272 (8.61%)	82 (2.60%)	29 (0.92%)
≥ 56	3177	299 (9.41%)	67 (2.11%)	241 (7.59%)	100 (3.15%)	25 (0.79%)
χ^2^		1.9433		93.3811		
*P*-value		0.1633		<0.0001		
Nation						
Other	529	55 (10.40%)	21 (3.97%)	64 (12.11%)	6 (1.13%)	6 (1.13%)
Han	29,178	3404 (11.67%)	807 (2.77%)	3931 (13.47%)	118 (0.40%)	162 (0.56%)
Hui	1360	173 (12.72%)	33 (2.43%)	192 (14.12%)	11 (0.81%)	3 (0.22%)
Kazak	1092	127 (11.63%)	31 (2.84%)	143 (13.10%)	10 (0.92%)	5 (0.46%)
Mongol	483	67 (13.87%)	12 (2.48%)	73 (15.11%)	4 (0.83%)	2 (0.41%)
Uygher	5080	452 (8.90%)	105 (2.07%)	516 (10.16%)	31 (0.61%)	10 (0.20%)
χ^2^		5.6903		37.043		
*P*-value		0.3375		<0.0001		
Year						
2009	1974	324 (16.41%)	84 (4.26%)	83 (4.20%)	33 (1.67%)	12 (0.61%)
2010	2322	447 (19.25%)	129 (5.56%)	494 (21.27%)	41 (1.76%)	41 (1.76%)
2011	1952	329 (16.85%)	87 (4.46%)	346 (17.73%)	44 (2.25%)	26 (1.33%)
2012	2352	385 (16.37%)	172 (7.31%)	470 (19.98%)	61 (2.59%)	26 (1.11%)
2013	1712	221 (12.91%)	200 (11.68%)	367 (21.44%)	39 (2.28%)	15 (0.88%)
2014	1483	375 (25.29%)	67 (4.52%)	389 (26.23%)	43 (2.90%)	10 (0.67%)
2015	3106	490 (15.78%)	68 (2.19%)	499 (16.07%)	41 (1.32%)	18 (0.58%)
2016	6657	725 (10.89%)	85 (1.28%)	758 (11.39%)	44 (0.66%)	8 (0.12%)
2017	10,693	476 (4.45%)	54 (0.51%)	486 (4.55%)	38 (0.36%)	6 (0.06%)
2018	5366	501 (9.34%)	34 (0.63%)	490 (9.13%)	30 (0.56%)	10 (0.19%)
χ^2^		426.35		160.84		
*P*-value		0.000		<0.0001

**Table 6:** Frequency and prevalence (%) of representatives of HPV grouped by age, nationality and year

	HPV16	HPV52	HPV53	HPV58
Age				
≤ 25	78 (4.36%)	59 (3.30%)	32 (1.79)	41 (2.29%)
26–30	182 (3.70%)	130 (2.64%)	61 (1.24%)	74 (1.50%)
31–35	227 (3.86%)	136 (2.31%)	65 (1.11%)	114 (1.94%)
36–40	313 (4.94%)	185 (2.92%)	98 (1.55%)	135 (2.13%)
41–45	283 (4.02%)	184 (2.61%)	92 (1.31%)	120 (1.70%)
46–50	178 (3.29%)	124 (2.29%)	81 (1.50%)	93 (1.72%)
51–55	88 (2.79%)	61 (1.93%)	56 (1.77%)	45 (1.42%)
≥ 56	113 (3.56%)	74 (2.33%)	35 (1.10%)	58 (1.83%)
Nation				
Other	17 (3.21%)	19 (3.59%)	2 (0.38%)	15 (2.84%)
Han	1107 (3.79%)	808 (2.77%)	413 (1.42%)	565 (1.94%)
Hui	58 (4.26%)	42 (3.09%)	19 (1.40%)	32 (2.35%)
Kazak	63 (5.77%)	8 (0.73%)	18 (1.65%)	17 (1.56%)
Mongol	22(4.55%)	14(2.90%)	8(1.66%)	6 (1.24%)
Uygher	195(3.84%)	62(1.22%)	60(1.18%)	45 (0.89%)
Year				
2009	140 (7.09%)	55 (2.79%)	35 (1.77%)	59 (2.99%)
2010	191 (8.23%)	76 (3.27%)	80 (3.45%)	84 (3.62%)
2011	157 (8.04%)	47 (2.41%)	32 (1.64%)	63 (3.22%)
2012	195 (8.29%)	98 (4.17%)	61 (2.59%)	78 (3.32%)
2013	167 (9.75%)	106 (6.19%)	71 (4.15%)	88 (5.14%)
2014	119 (8.02%)	68 (4.59%)	35 (2.36%)	59 (3.98%)
2015	108 (3.48%)	106 (3.41%)	42 (1.35%)	51 (1.64%)
2016	145 (2.18%)	221 (3.31%)	87 (1.31%)	64 (0.96%)
2017	103 (0.96%)	94 (0.88%)	36 (0.34%)	65 (0.61%)
2018	120 (2.24%)	80 (1.49%)	40 (0.74%)	62 (1.16%)

From 2009 to 2013, the HPV infection rate fluctuated but did not change much. It peaked in 2014 and then fell significantly, reached the bottom point in 2017 and rose slightly in 2018 (Fig. 4a). In 10 years, the single infection was higher than multiple infection, and the change trend of single infection was the same as the overall change trend. Multiple infection gradually increased from 2009 to 2013 and reached a peak, and then gradually decreased (Fig. 4b). Pure HR infection dominated in 10 years. Except 2009, the change trend was similar to the overall change trend. Pure LR and mixed infection did not change significantly with time (Fig. 4c). The variation trend of several typical genotypes of HPV was similar to the overall variation trend (Fig. 4d).

## Discussion

Xinjiang is located in the northwest of China, the economic underdevelopment, cervical cancer burden is higher than that of economic developed areas of China, so primary prevention of cervical cancer in Xinjiang is particularly important. If the vaccination could effectively reduce the incidence of HSIL and cervical cancer, it would not only benefit women’s health, but also save a lot of medical expenses for the country. Research in the prevalence and genotypes distribution of HPV in different areas and periods is very important for cervical cancer screening and the evaluation of the efficacy of HPV vaccines for females. In the last 20 years, major efforts have been made worldwide to generate epidemiological data on cervical HPV-DNA. And our research is one of the largest studies in China for HPV detection and the first large sample study conducted in Xinjiang.

Due to differences in regions, populations and HPV DNA testing methods, the reported results of global HPV distribution varies from study to study. Globally, the region with the highest HPV infection rate is Sub-Saharan Africa (24.0%), Eastern Europe (21.4%), Latin America (16.1%), and Southeastern Asia (14%) successively [20]. It was reported that the overall prevalence of HR-HPV varied from 9.9–27.5% in China. In our study, the women’s overall prevalence of HPV was 14.02%, which was lower than the average global level, higher than that of many other cities or areas in China, such as Beijing, Guangdong and Shandong [21–23], and lower than that of Shanghai, Tianjin and Chongqing [24, 25]. Currently, researchers speculated that the variable HPV prevalence in China occurred because of the large Chinese population and its territories [26]. Meanwhile, the level of economic development and population migration also led to HPV distribution differences among regions. As mentioned above, Beijing, as the political, cultural and economic center of China, has good medical conditions and the lowest HPV infection rate. The economic development level of Shanghai was also very high, but because of a large number of foreign population, the city was expanding, the population composition was complex, which led to the highest HPV infection rate. Although there were regional differences in HPV infection rates, they were all dominated by high-risk HPV infections. In our study, compared with pure LR and mixed HPV infection, the overall prevalence of pure HR-HPV occupied the dominant position. After stratification by age, nationality and year respectively, the tendency of HR-HPV was still higher, which was consistent with the results of above study (Table 5 and Figs. 2, 3, and 4).

In this research, HPV 16 (3.79%), 52 (2.47%), 58 (1.76%), 53 (1.35%), and 31 (0.72%) were the five most common HPV genotypes among females while HPV 16, 52, 58, 33, 18 were regarded as the most prevalent genotypes in China [27]. Nevertheless, different cities might show diversity with respect to HPV genotypes among females: HPV 16, 58, 33 were the top three HPV genotypes in Beijing, and HPV 16, 53, 52 were the top three HPV genotypes in northern Xinjiang [28]. Zhao et al. [29] reported that HPV52 infections were more common among healthy individuals, whereas HPV58 were linked to cervical cancer. HPV 53 was nonvaccine genotype and now recognized as one of the four “emergent” genotypes, with a possible role in oncogenesis [30]. Based on the above data, we could summarize the following points: ① HPV 16 was the most prevalent genotype of women in this study, which was consistent with many other domestic and abroad researches; ②HPV18, apart from HPV16, was also important for cervical carcinogenesis. However, in our present study, it was the ninth most common genotype, and the infection rate was only 0.65%; ③ Although both HPV 52 and 58 were all common among Asian populations, the significance of the two genotypes remained unknown, which needed further investigation. ④ The prevalence of human papillomavirus 53 (HPV 53) in Xinjiang was relatively prominent, ranked the fourth among the five major genotypes in this region. These findings indicated that in addition to HPV16, 18, 52, 58, the HPV vaccine of HPV53 genotype should also be included in Xinjiang. Current screening strategies use HPV16 or 18 positivity as indicators for further colposcopy and cervical biopsy. Nonetheless, whether this practice is applicable to China and Xinjiang remains to be potential studied.

Information on the distribution of HPV infection at different ages was critical for the design of age-specific prophylactic HPV vaccines. Regarding the HPV prevalence in different age groups, there were similarities and differences in scholars’ research. What many studies had in common was that the first peak appeared at a younger age group (just after beginning sexual relations). In some regions, a modest second peak was observed at age >40 years or >45 years or >55 years or >60 years. In some regions, this second peak was not observed. To sum up, age-specific HPV distribution presented as either a bimodal curve (including the “U” curve) or a unimodal distribution skewed to the left [20, 22, 26]. In developed countries and economically developed areas of developing countries, the distribution of age-specific HPV was dominated by the “U” curve. In this study, age-specific HPV distribution presented as a bimodal curve. The first peak of HPV infection occurred in ≤25 age group (20.78%). However, the second peak emerged in 36–40 age group (15.45%), which was younger than the data provided in the Bruni study. And the HPV infection rate gradually declined in>40 age groups, which was different from many developed countries in Europe and America and some cities in China, but the same as some Asian countries. Young females were sensitive to HPV soon after the beginning of sexual activity because of immature immune protection, nevertheless, most cases of HPV infection were usually temporary and would be cleared rapidly [31]. In this study, we didn’t observe an increase trend in HPV infection rate in perimenopause or postmenopausal women, which was mainly caused by the following three factors. First, cervical cancer screening coverage was insufficient, and many women in this age group didn’t accept screening. Second, in this age group of the awareness of women health was weakly, they thought that they did not need cervical cancer screening after menopause. Third, many premenopausal and postmenopausal women had less frequent sexual activity due to cultural differences. Althoff et al. [32] suggested that the reasons for the second peak of HPV prevalence with age might also be related to body mass index, ethnicity, sexual behavior, HPV type and variants, host susceptibility and individual screening practices, nonetheless, the exact mechanisms for the increase in HPV prevalence still remained unclear and needed further analysis.

International correlation between the prevalence of HR-HPV infection in the general population and its cervical cancer burden has been proven [33]. It is widely believed that the regions with high HPV prevalence were the ones with the highest cervical cancer incidences, and the regions with lower prevalence had the lowest incidences. However, we found the opposite result in our study, the incidence of cervical cancer in Uygur women in Xinjiang was high [2], but compared with other ethnic groups in Xinjiang, the uygur ethnic group had the lowest HPV infection rate(8.90%). Some studies from India were similar to this study. Eastern Europe was the opposite, which had a high HPV prevalence (21.4%) but a relatively low incidence. Scholars reported [34] that Dai women HPV infection rate (9.9%) was low because of good living environment and health conditions, in contrast, the majority of individuals in the other ethnic population (for example, HPV infection rate of Tibetan women was 27.4%) were illiterate, with a high fertility rate, they mainly worked in agricultural farming, and lived in remote mountainous areas with poor sanitary conditions. Unlike this condition in our study, although many uygur patients had the similar living and health conditions as Tibetans, they had a lower rate of HPV infection. These differences fully demonstrated that the occurrence of cervical cancer was not only related to HPV infection, but also closely related to genetic susceptibility.

In this study, from 2009 to 2014, the HPV infection rate maintained at a high level (20.62% in 2009) and reached peak in 2014 (29.80%), which might be attributed to the change of cervical cancer screening strategy. In 2011 [35], the recommended screening strategies were cytology and cotesting (cytology in combination with HPV testing). HPV testing was a method for further stratification of women with cytological abnormalities. Therefore, more females were being tested for HPV, leading to an increase in HPV infection rate. HPV-positive rate showed a downward trend from 2014 to 2017 (from 29.80% in 2014 to 4.96% in 2017). Similarly, single, multiple, HR-HPV and several major genotypes infection (16, 52, 58, 53) were in line with the downward trend. The same phenomenon was observed in the studies of Zeng, Z. et al. [36] and Chen X. et al [37] The decline in prevalence might because of the following reasons: (1) With the increasing attention to cervical cancer, the approval for launch of precancerous lesions and HPV vaccine in China in 2017, more and more females (for example, women who didn’t pay attention to screening for cervical cancer previously, women who had negative cervical cytology results, women who had vaccination intentions, and women who were asymptomatic) attended HPV screening, which resulted in that the prevalence of HPV tended to be natural infection rate. (2) Some HPV-positive women were tested for HPV over the next few years, which turned negative, possibly due to immune system clearance and local physical treatment and conization.

The results of this study showed that HPVl6 and 52 were common infection genotypes in Chinese population, which was consistent with the results of most cross-sectional studies in China [27–29]. However, further studies also found that the infection rate of HPV16 gradually decreased during the screening period, and HPV52 became the main infection type, showing that the infection rate of HPVl6 was up to 8.04% in the total population in 2011, which was much higher than common types such as HPV52 (2.41%) and 58 (3.22%). In 2015, the infection rate of HPVl6 and 52 in the population was almost the same (both 3.40%), while the HPV52 type (3.31%) was higher than HPVl6 type (2.18%) and became the dominant type in 2016. The similar results appeared in the study of Qiao Youlin et al. [38]. Our study also indicated that the infection rate of HPV16 and 52 increased suddenly after 56 years old. This was another characteristic change accompanied by HPVl6 and 52 dominance type transformation, suggesting that both genotypes might have common influencing factors.

The major strengths of this study were the large number of patients, in addition, the study was a decade long, so we could see a yearly trend of HPV infection in Xinjiang. However, several limitations should be acknowledged. First, the data of HPV infection were not further confirmed with cytopathology and histopathology, resulting in the effectiveness of HPV screening could not be accurately assessed. Especially, we didn’t have any additional information from the women testing that the HR-HPV was positive but the liquid-based cytology was negative, hindering a more comprehensive evaluation of the role what HPV testing played in screening in this population. Furthermore, a follow-up study should be done to track changes in genotype, cervical pathology and cytology as there was a close relationship between cervical carcinoma and long-term persistent HR-HPV infections. Finally, the results relied on samples referred to our laboratory for diagnostic purposes. Thus, these data cannot be considered nationally representative because the Xinjiang women included in the study might not be a representative sample of all the China women living in the country.

## Conclusions

In conclusion, the most significant findings in this survey consisting 37,722 samples in Xinjiang Province are as follows: (1) the prevalence of HPV infection showed variations by age, nationality and year. (2) HPV 16, 52, 58, 53, 31 were always the major genotypes, although the rank varied according to the age, nationality and year; particular attention should be paid to the high prevalence of non-vaccine genotypes (e.g. HPV53). (3) Although the prevalence of HPV had decreased in recent years, the HR-HPV infection and the changes of the main infection type cannot be ignored (for example, the infection rate of HPV52 was close to HPV16’s and even higher than that of HPV16). Our results would provide guidance for primary screening and vaccination program for cervical cancer.

## Data Availability

Our data will not be shared to protect the participants’anonymity.

## Abbreviations

CC: Cervical cancer
EQA: External Quality Assessment
HPV: Human papillomavirus
HR-HPV: High-risk human papillomavirus
HSIL: High grade cervical lesions
IQC: Internal Quality Control
LR-HPV: Ligh-risk human papillomavirus
WHO: The world health organization

## References

[1] Bray F, Ferlay J, Soerjomataram I (2018). Global cancer statistics 2018: GLOBOCAN estimates of incidence and mortality worldwide for 36 cancers in 185 countries. CA Cancer J Clin.

[2] Mijit F, Ablimit T, Abduxkur G, Abliz* G (2015). Distribution of human papillomavirus (HPV)genotypes detected by routine pap smear in Uyghur-Muslim women from Karasay township Hotan (Xinjiang, China). J Med Virol.

[3] Trottier H, Franco EL (2006). Human papillomavirus and cervical cancer: burden of illness and basis for prevention. Am J Manag Care.

[4] Cogliano V (2005). Carcinogenicity of human papillomaviruses. Lancet Oncol.

[5] Wolday D, Derese M (2018). HPV genotype distribution among women with normal and abnormal cervical cytology presenting in a tertiary gynecology referral Clinic in Ethiopia. Infect Agent Cancer.

[6] Munoz N, Bosch FX, de Sanjose S, Herrero R, Castellsague X, Shah KV (2003). Epidemiologic classification of human papillomavirus types associated with cervical cancer. N Engl J Med.

[7] Agorastos T, Chatzistamatiou* K, Katsamagkas T (2015). Primary screening for cervical Cancer based on high-risk human papillomavirus (HPV) detection and HPV 16 and HPV 18 genotyping. PLoS One.

[8] Piana A, Sotgiu G, Castiglia P, Pischedda S, Cocuzza C, Capobianco G (2011). Prevalence and type distribution of human papillomavirus infection in women from North Sardinia, Italy. BMC Public Health.

[9] de Oliveira CM, Aguiar LS, Genta ML, Alves VA, Levi JE (2011). HPV-11 associated metastatic cervical cancer. Gynecol Oncol Case Rep.

[10] Durst M, Gissmann L, Ikenberg H, zur HH (1983). A papillomavirus DNA from a cervical carcinoma and its prevalence in cancer biopsy samples from different geographic regions. Proc Natl Acad Sci U S A.

[11] Walboomers JM, Jacobs MV, Manos MM, Bosch FX, Kummer JA, Shah KV, Snijders PJ, Peto J, Meijer CJ, Muoz N (1999). Human papillomavirus is a necessary cause of invasive cervical cancer worldwide. J Pathol.

[12] Loud J, Murphy J (2017). Cancer screening and early detection in the 21st century. Semin Oncol Nurs.

[13] Brismar-Wendel S, Froberg M, Hjerpe A, Andersson S, Johansson B (2009). Age-specific prevalence of HPV genotypes in cervical cytology samples with equivocal or low-grade lesions. Br J Cancer.

[14] Niyazmetova L, Aimagambetova G, Stambekova N (2017). Application of molecular genotyping to determine prevalence of HPV strains in pap smears of Kazakhstan women. Int J Infect Dis.

[15] Seraceni S, Campisciano G, Contini C, Comar M (2016). HPV genotypes distribution in chlamydia trachomatis co-infection in a large cohort of women from north-East Italy. J Med Microbiol.

[16] Sasagawa T, Maehama T, Ideta K, Irie T, Fujiko Itoh JHSG (2016). Population-based study for human papillomavirus (HPV) infection in young women in Japan: a multicenter study by the Japanese human papillomavirus disease education research survey group (J-HERS). J Med Virol.

[17] Dareng EO, Ma B, Famooto AO (2016). Prevalent high-risk HPV infection and vaginal microbiota in Nigerian women. Epidemiol Infect.

[18] de Sanjose S, Quint WG, Alemany L, Geraets DT, Klaustermeier JE, Lloveras B (2010). Human papillomavirus genotype attribution in invasive cervical cancer: a retrospective cross-sectional worldwide study. Lancet Oncol.

[19] Wang J, Tang D, Ma C, et al. Genotype distribution and prevalence of human papillomavirus among women with cervical cytological abnormalities in Xinjiang, China. Hum Vaccin Immunother. 2019;8:1–8. 10.1080/21645515.2019.1578598. 10.1080/21645515.2019.1578598 PMC6746534 30735478

[20] Hong P, Wang PC, Zhang YX, Han P (2014). Prevalence and subtype distribution of HPV infection among women in Beijing urban area and their correlation with age. Zhonghua Nan Ke Xue.

[21] Yuan X, Yang Y, Gu D, Liu H, Yang H, Wang M (2011). Prevalence of human papillomavirus infection among women with and without normal cervical histology in Shandong Province, China. Arch Gynecol Obstet.

[22] Huang Y, Lin M, Luo ZY, Li WY, Zhan XF, Yang LY (2013). Low prevalence of HPV in male sexual partners of HR-HPV infected females and low concordance of viral types in couples in eastern Guangdong. Asian Pac J Cancer Prev.

[23] Chen X, Wallin KL, Duan M, Gharizadeh B, Zheng B, Qu P (2015). Prevalence and genotype distribution of cervical human papillomavirus (HPV) among women in urban Tianjin, China. J Med Virol.

[24] Zhang C, Zhang C, Huang J, Wu Z, Mei X, Shi W (2018). Prevalence and genotype distribution of human papillomavirus among females in the suburb of Shanghai, China. J Med Virol.

[25] Wang R, Guo X-l, Bea G, Wisman A (2015). Nationwide prevalence of human papillomavirus infection and viral genotype distribution in 37 cities in China. BMC Infect Dis.

[26] Lorenzon L, Terrenato I, Dona MG (2014). Prevalence of HPV infection among clinically healthy Italian males and genotype concordance between stable sexual partners. J Clin Virol.

[27] Liu XX, Fan XL, Yu YP, Ji L, Yan J, Sun AH (2014). Human papillomavirus prevalence and type-distribution among women in Zhejiang Province, Southeast China: a cross-sectional study. BMC Infect Dis.

[28] Wang L, Wang P, Ren Y (2016). Prevalence of high-risk human papillomavirus (HR-HPV) genotypes and multiple infections in cervical abnormalities from northern Xinjiang, China. PLoS One.

[29] Zhao FH, Tiggelaar SM, Hu SY, Xu LN, Hong Y, Niyazi M (2012). A multi-center survey of age of sexual debut and sexual behavior in Chinese women: suggestions for optimal age of human papillomavirus vaccination in China. Cancer Epidemiol.

[30] Kocjan BJ, Seme K, Mocilnik T, Jancar N, Vrtacnik-Bokal E, Poljak M (2007). Genomic diversity of human papillomavirus genotype 53 in an ethnogeographically closed cohort of white European women. J Med Virol.

[31] Rodriguez AC, Schiffman M, Herrero R (2008). Rapid clearance of human papillomavirus and implications for clinical focus on persistent infections. J Natl Cancer Inst.

[32] Althoff KN, Paul P, Burke AE, Viscidi R, Sangaramoorthy M, Gravitt PE (2009). Correlates of cervicovaginal human papillomavirus detection in perimenopausal women. J Women's Health (Larchmt).

[33] Maucort-Boulch D, Franceschi S, Plummer M, IARC HPV Prevalence Surveys Study Group (2008). International correlation between human papillomavirus prevalence and cervical cancer incidence. Cancer Epidemiol Biomark Prev.

[34] Baloch Z, Yasmeen N, Li Y (2017). Prevalence and risk factors for human papillomavirus infection among Chinese ethnic women in southern of Yunnan, China. Braz J Infect Dis.

[35] Huh WK, Ault KA, Chelmow D (2015). Use of primary high-risk human papillomavirus testing for cervical cancer screening: interim clinical guidance. Gynecol Oncol.

[36] Zeng Z (2016). Prevalence of high-risk human papillomavirus infection in China: analysis of 671,163 human papillomavirus test results from China’s largest College of American Pathologists-Certified Laboratory. Am J Clin Pathol.

[37] Chen X, Xu H, Xu W (2017). Prevalence and genotype distribution of human papillomavirus in 961,029 screening tests in southeastern China (Zhejiang Province) between 2011 and 2015. Sci Rep.

[38] Youlin Q, Li D, Shangying H (2017). Changes in genotype prevalence of human papillomavirus over 10-year follow-up of a cervical cancer screening cohort. Chi J Epidemiol.

